# Sedentary Lifestyle Leads to Increased Osteoarthritis Among Diabetic Patients

**DOI:** 10.7759/cureus.110168

**Published:** 2026-06-03

**Authors:** Nequesha S Mohamed, Ronald Delanois, Johannes F Plate, Jeffrey S Willey

**Affiliations:** 1 Department of Orthopaedics, Wake Forest School of Medicine, Winston-Salem, USA; 2 Center for Joint Preservation and Replacement, Sinai Hospital of Baltimore, Baltimore, USA; 3 Department of Orthopaedic Surgery, University of Pittsburgh, Pittsburgh, USA; 4 Department of Radiation Oncology, Wake Forest School of Medicine, Winston-Salem, USA

**Keywords:** diabetes, joint pain, osteoarthritis, physical activity, sedentary lifestyle

## Abstract

Introduction

As a disease of aging, osteoarthritis affects many Americans. It can be coincident with diabetes, which can increase the risk of joint degradation needing joint replacement. A lack of physical activity, often concurrently present in type 2 diabetic patients, may also increase osteoarthritis risk. The resulting sedentary lifestyle could exacerbate arthritic changes in this population. We compare 5-year incidences of osteoarthritis, joint pain, and joint replacement between matched sedentary and non-sedentary diabetic patients.

Methods

Diabetic patients from 2010-2018 were identified in a national database, and sedentary patients were propensity-matched 1:1 to non-sedentary patients (n=2,238 pairs). Osteoarthritis and joint pain within 5 years were compared utilizing chi-square. A sub-cohort of osteoarthritic diabetic patients with/without sedentary lifestyle was also propensity-matched (n=1,033 pairs), comparing total knee arthroplasty (TKA) and total hip arthroplasty (THA) incidences within 5 years.

Results

Compared to non-sedentary diabetic patients, sedentary diabetics had more osteoarthritis (29.6% vs 38.9%, p<0.001) and significantly higher 5-year incidences of joint pain (27.3% vs 36.0%, p<0.001). For diabetic patients with osteoarthritis, non-sedentary and sedentary patients had similar rates of TKA (2.7% vs 4.1%, 1.5% vs 1.7%, p=0.114) or THA (p=0.726) at 5 years.

Conclusion

Lack of physical activity among diabetic patients may exacerbate both osteoarthritis and joint pain within 5 years. Conversely, lack of physical activity did not increase joint replacements. Osteoarthritic changes may be augmented with a sedentary lifestyle, but are not severe enough to necessitate arthroplasty. Sedentary diabetic patients represent a target population for early intervention and recovery exercises to improve joint function and prevent osteoarthritis progression.

## Introduction

Osteoarthritis (OA), as one of the most common disabilities of aging, has risen over 48% in the past 30 years globally, with over 52.5 million adults afflicted in the United States alone [1,2]. Many patients experience progressive disease, which necessitates a joint replacement later in life. Diabetic patients are particularly susceptible to joint degeneration, as a complex interaction of factors predisposes them to a greater risk of osteoarthritis and cartilage degradation [3]. With over 34 million diabetic patients in the United States, the coincidence of these two diseases in the elderly population is growing due to shared risk factors and high prevalence [4]. Aging is the most common risk factor for diabetes and osteoarthritis, though obesity has a strong connection to both as well. Furthermore, nearly half of diabetic patients suffer from some type of arthritis, underscoring the relationship these two diseases share [5,6].

Diabetes is a metabolic disorder that disrupts several body functions and pathways through hyperglycemia and hyperinsulinemia, producing inflammatory byproducts as a result [3,7]. Similarly, osteoarthritis is an inflammatory disease typically localized to the joint and is due to mechanical wear and tear [8]. However, there is increasing evidence that systemic inflammation can lead to an increase in inflammatory markers in the joint, subsequently inducing osteoarthritis [9,10]. As a condition that causes a spread in inflammation globally, diabetes has had growing links to osteoarthritis and is thought to independently increase the risk of disease/progression [3,11,12]. Furthermore, patients suffering from both diabetes and osteoarthritis can have an increased severity of joint pain, even after controlling for confounders [13].

Physical activity has been studied in both conditions and can help slow progression and improve symptoms [14-17]. However, independently, both diabetic and osteoarthritic patients are more likely to be sedentary or have a lower physical activity level, which may exacerbate their symptoms inadvertently, including joint pain [18,19]. While physical activity has been demonstrated to improve both diabetic and osteoarthritic outcomes, there is a paucity of data regarding how the lack of physical activity can affect joint pain and subsequent joint replacement in this population.

A sedentary lifestyle, which has a lack of physical activity as a defining feature, may contribute to increasing incidences of osteoarthritis and joint pain. The link between diabetes and OA has been assessed, but reports assessing the lack of physical activity in the setting of both are lacking. Thus, this study will investigate osteoarthritis outcomes in patients with diabetes who do and do not have a sedentary lifestyle. Specifically, we compare the 5-year incidences of 1) osteoarthritis, 2) joint pain, and 3) joint replacement between matched sedentary and non-sedentary diabetic patients.

This article was previously presented as a meeting abstract at the 2022 International Cartilage Repair Society (ICRS) World Congress Meeting on April 14, 2022.

## Materials and methods

Database

This prospectively collected, retrospectively reviewed Level III study utilized the PearlDiver Supercomputer (PearlDiver Technologies, Inc., Fort Wayne, USA). This database is publicly available and contains deidentified health records from over 122 million patients across the country [20]. Patients can be longitudinally tracked over several years with unique patient identifiers. This database contains information such as Current Procedural Terminology (CPT), International Classification of Diseases (ICD) 9 and 10 procedural and diagnosis codes, demographic information, medication information, and claim reimbursement information. This study was exempt from Institutional Review Board Review.

Patient population

The database was searched for all patients who had a diagnosis of type 2 diabetes between January 2010 and July 2018, utilizing PearlDiver’s comorbidity identifier. Inclusion criteria were all patients with a diagnosis of “lack of physical exercise” (ICD 9: V69.0, ICD 10: Z72.3), which was utilized to identify patients with a sedentary lifestyle among this cohort. Exclusion criteria were any patients who did not have the exercise ICD codes, and anyone outside the 8-year study range. The remaining patients were then propensity score-matched on demographics, including age, sex, Charlson Comorbidity Index (CCI), tobacco abuse, and obesity status, to non-sedentary diabetic patients, yielding 2,238 matched pairs. A sub-cohort of diabetic patients who were diagnosed with osteoarthritis was also matched 1:1 based on a sedentary lifestyle using demographics, resulting in 1,033 matched pairs.

Outcomes

Diagnoses of osteoarthritis of the knee and hip, as well as joint pain, specifically in the hip and lower leg, were collected within 5 years of the diagnosis of diabetes. Additionally, in diabetic patients with osteoarthritis, the incidence of total knee arthroplasty (TKA) and total hip arthroplasty (THA) was collected and compared between sedentary and non-sedentary patients.

Statistical analysis

Categorical variables were compared through Chi-Square analyses, while continuous variables were assessed through Student t-tests. Significance was set to p<0.05 for all comparisons. All analyses were performed with RStudio analysis software (R Foundation for Statistical Computing, Vienna, Austria) through the PearlDiver Supercomputer.

## Results

Between sedentary diabetic patients and non-sedentary diabetic patients, there were no significant differences in age (56 vs. 56 years, p=0.918), sex (male: 35.6 vs. 35.6%, p>0.999), CCI (p>0.999), obesity (53.2 vs. 53.2%, p>0.999), and tobacco abuse (18.7 vs. 18.7%, p>0.999) (Table 1).

**Table 1:** Demographics and Outcomes of Diabetic Patients Data are presented as n (%), unless otherwise specified. Tests: T-test (age), Chi-square (sex, obesity, tobacco, total knee arthroplasty, total hip arthroplasty) SD: standard deviation

	Sedentary Diabetic Patients (n=2,238)	Non-Sedentary Diabetic Patients (n=2,238)	Test Statistic	P-value
Age, mean (SD) years	56.1 (14.9)	56.1 (14.8)	-0.10257	0.918
Sex			0	0.999
Male	796 (35.6)	796 (35.6)		
Female	1,442 (64.4)	1,442 (64.4)		
Obesity Status			0	0.999
Non-obese	1,048 (46.8)	1,048 (46.8)		
Obese	1,190 (53.2)	1,190 (53.2)		
Tobacco Abuse			0	0.999
No	1,820 (81.3)	1,820 (81.3)		
Yes	418 (18.7)	418 (18.7)		
Osteoarthritis at 5 years			42.922	<0.001
No	1,367 (61.1)	1,576 (70.4)		
Yes	871 (38.9)	662 (29.6)		
Joint Pain at 5 years			38.45	<0.001
No	1,432 (64.0)	1,626 (72.7)		
Yes	806 (36.0)	612 (27.3)		

This signifies that the propensity score matching was successful, and patients can be compared without worry that they are unbalanced. Sedentary patients had a significantly higher 5-year incidence of osteoarthritis compared to non-sedentary patients (38.9 vs. 29.6%, p<0.001). Sedentary patients also had a significantly higher 5-year incidence of joint pain (36.0 vs. 27.3%, p<0.001). This suggests that when factors related to diabetes are accounted for, a lack of physical exercise results in significantly higher rates of osteoarthritis, as well as significantly higher rates of reported joint pain, which can typically be the precursor to diagnosed osteoarthritis, suggesting physical activity to be important in regulating joint degradation.

Within the sub-cohort of diabetic patients with osteoarthritis, no significant differences were observed between the groups in age (61 vs. 61 years, p=0.994), sex (male: 30.9 vs. 30.9%, p>0.999), CCI (p>0.999), obesity (57.0 vs. 57.0%, p>0.999), and tobacco abuse (20.1 vs. 20.1%, p>0.999) (Table 2).

**Table 2:** Demographics and Outcomes of Diabetic Patients With Osteoarthritis Data are presented as n (%), unless otherwise specified. Tests: T-test (age), Chi-square (sex, obesity, tobacco, total knee arthroplasty, total hip arthroplasty) SD: Standard Deviation

	Sedentary Diabetic Patients with Osteoarthritis (n=1,033)	Non-Sedentary Diabetic Patients with Osteoarthritis (n=1,033)	Test Statistic	P-value
Age, mean (SD) years	61.3 (10)	61.3 (10.9)	0.0081267	0.994
Sex			0	0.999
Male	319 (30.9)	319 (30.9)		
Female	714 (69.1)	714 (69.1)		
Obesity Status			0	0.999
Non-obese	444 (43.0)	444 (43.0)		
Obese	589 (57.0)	589 (57.0)		
Tobacco Abuse			0	0.999
No	825 (79.9)	825 (79.9)		
Yes	208 (20.1)	208 (20.1)		
Total Knee Arthroplasty at 5 years			2.499	0.114
No	991 (95.9)	1,005 (97.3)		
Yes	42 (4.1)	28 (2.7)		
Total Hip Arthroplasty at 5 years			0.12318	0.726
No	1,015 (98.3)	1,018 (98.5)		
Yes	18 (1.7)	15 (1.5)		

The propensity score matching was still valid and created perfectly matched groups for comparison, removing diabetes and osteoarthritis as potential confounders for this portion of the analysis. When sedentary patients were compared to non-sedentary patients, there were no significant differences observed in the occurrence of TKA over 5 years (4.1 vs. 2.7%, p=0.114). Additionally, there were no significant differences in the 5-year occurrence of THA (1.7 vs. 1.5%, p=0.726). The results of this analysis demonstrate that within the osteoarthritis group, the incidence of patients undergoing total joint replacement was not increased for sedentary patients, suggesting that the joint pain they experience may not be significant enough to warrant surgical treatment within 5 years. A lack of physical exercise does not appear to influence the decision for diabetic patients with arthritis to undergo joint replacement.

## Discussion

The co-incidence of diabetes and osteoarthritis has increased in prevalence in recent years, likely due to an overlap in risk factors such as obesity and abnormal metabolism [5,6]. Many diabetic patients subsequently develop osteoarthritis due to generalized inflammation in the body [9,10]. Afflicted patients are likely to be more sedentary, which can potentially promote disease progression and lead to worse outcomes both separately and in combination [18,19].

This study investigated the 5-year incidence of osteoarthritis and joint pain in diabetic patients with and without documented lack of physical activity, in addition to the 5-year incidence of TKA and THA in these osteoarthritic patients. We found that sedentary diabetic patients had a significantly higher incidence of both osteoarthritis and joint pain within 5 years, but the diabetic patients with osteoarthritis did not have a higher incidence of TKA or THA. This could suggest that while sedentary behaviors promote the development of osteoarthritis and related symptoms, a sedentary lifestyle does not lead to increases in definitive joint treatments. It may take longer to degrade the joint, though early changes can be seen. Sedentary patients may perceive a significant limitation of their sedentary lifestyle compared to more active patients.

Several studies have reported on the orthopedic symptoms that diabetes has an impact on. A retrospective study of 819 patients by Alenazi et al. [13] examined type 2 diabetes and pain severity in localized osteoarthritis. After controlling for confounding factors, diabetes was associated with increased severity of joint pain (p=0.014), and higher HbA1c values indicated higher pain severity (p=0.029). A study by Eitner et al. [21] also assessed knee pain intensity in 70 TKA patients with and without diabetes. They found that the diabetic patients had a higher pain score prior to surgery when compared to the non-diabetic patients (p<0.001), and that the diabetics had higher concentrations of interleukin (IL)-6, an inflammatory cytokine, in their synovial fluid (p=0.003).

Similarly, Schett et al. [22] performed a population study of 927 patients to determine if type 2 diabetes predicted severe osteoarthritis requiring arthroplasty in an age and sex matched cohort. They found arthroplasty rates to be significantly higher in diabetic patients (p<0.001), with diabetes as an independent risk factor after controlling for confounders (p=0.023). Diabetic patients also had higher pain scores prior to their surgeries as well (p<0.001). These studies establish the foundation that osteoarthritis severity is typically increased in diabetes and can be exacerbated in those without good control.

Evidence has demonstrated the beneficial effects of exercise on the management of diabetes and its symptoms. A meta-analysis by Umpierre et al. [23] assessed the associations between exercise regimens and changes in A1c levels in diabetic populations. Their analysis found that a structured exercise program lowered HbA1c levels 0.67% (95% confidence interval (CI), -0.84% to -0.49%), and when physical activity advice was given with dietary advice, HbA1c significantly decreased (-0.58%; 95% CI, -0.74% to -0.43%).

In a randomized controlled trial by Sigal et al. [24], HbA1c levels were measured in 251 adults with type 2 diabetes who underwent aerobic training, resistance training, or a combination, and compared to a sedentary control group. Aerobic exercise resulted in a 0.51% decrease in HbA1c (95% CI, -0.87 to -0.14) and resistance resulted in a 0.38% decrease (CI, -0.72 to -0.22) when compared to sedentary controls, while combination exercise lowered HbA1c by 0.46% and 0.59% compared to aerobic and resistance alone (CI, -0.83 to -0.09 and CI, -0.95 to -0.23). This demonstrates the beneficial effects exercise can have on glycemic control, which can help reduce inflammation.

Karstoft and Pedersen [25] reviewed literature on diabetes and exercise and discussed the anti-inflammatory effects exercise can have, as it can help increase muscle-derived IL-6, which stimulates IL-1 receptor antagonist and inhibits tumor necrosis factor-α to suppress inflammation, contrary to the synovial fluid-derived IL-6 mentioned earlier. The effects of exercise are multifold in diabetic patients, and a lack of exercise can be detrimental to their health.

Exercise for the management of osteoarthritis has been effective and is becoming an international guideline for nonoperative treatment, though there is still no consensus on the optimal exercise to perform; rather, any form seems to provide some positive effects. Goh et al. [26] performed a meta-analysis of randomized controlled trials to evaluate the effects of exercise on pain, function, performance, and quality of life. They determined that exercise significantly improved pain (effect size (ES) 0.56, 95% CI 0.44-0.68), functional outcomes (0.50, 0.38-0.63), performance metrics (0.46, 0.35-0.57), and quality of life measures (0.21, 0.11-0.31), though these effects were limited to about 2 months. The benefits were also most pronounced in younger patients with knee osteoarthritis who were not waiting to undergo replacement. This could be due to the higher activity levels younger people typically display, though any patients who reduce their movement could suffer from worsened symptoms.

A study of 84 hip and knee osteoarthritis patients by de Groot et al. [27] compared physical activity through movement to matched healthy controls. Their analysis revealed significantly lower movement activity levels in the osteoarthritis groups compared to control patients (p<0.05). Increasing age and body mass index were shown to negatively impact movement activities on regression analyses (beta=-0.29 and beta=-0.25, respectively). Low levels of movement predispose patients to worse outcomes as well.

Dunlop et al. [28] analyzed a cohort of 5,715 adults aged 65+ years who had arthritis. Though a fifth of the cohort had some baseline limitations in function, there was a further functional decline in another 13.6%, with lack of regular vigorous physical activity resulting in nearly double the odds of functional decline (adjusted odds ratio 1.9, 95% CI 1.5-2.4). In those who engaged in regular physical activity, this decline was reduced by up to 32%. Physical activity is important for pain reduction in osteoarthritis, but also for functional management, and without it, decline can soon be seen, as our study corroborates.

Strengths and limitations of the study

This study has several strengths, including establishing the link between a sedentary lifestyle and the development of osteoarthritis in the diabetic population, which has yet to be fully elucidated. It also allows comparisons to be made over the midterm period of 5 years to compare the development of joint pain and replacements.

There are a few limitations that exist in this study, though. First, there was no way to quantify the severity of diabetes in this population, such as hemoglobin A1c (HbA1C). This database contains a wealth of information, but does not provide all the desired data points, such as some lab values. However, it does contain enough to provide a general estimate of many conditions, like diabetes incidence. Second, the time to diagnosis of osteoarthritis or joint pain could not be determined in this database. This could have been helpful in determining how quickly these conditions develop, but it is still useful to know the number of diagnoses that occur within a given time period, as it gives an idea of how soon some conditions develop.

Finally, using the diagnosis code for “lack of physical exercise” may be an equivocal proxy for a sedentary lifestyle. The diagnosis may be recorded infrequently in a patient’s chart and could apply to patients who have alternate reasons for not being able to move. However, it is a billable code that can have serious health implications, as indicated by the results of this study. Additionally, the propensity score matching performed in this study helps to equalize many of the variables that could construe major differences in populations and ensure the validity of the comparisons made. The limitations of this study do not preclude its value to the literature in documenting the joint problems that exist for diabetic patients who do not exercise.

## Conclusions

Our report revealed that a lack of physical activity among diabetic patients led to a significantly higher incidence of both osteoarthritis and joint pain within 5 years. Conversely, a lack of physical activity among diabetic patients with osteoarthritis did not result in an increase in TKA or THA. This could suggest that osteoarthritic changes are augmented with a sedentary lifestyle, but are not severe enough to necessitate a joint replacement in the short term. This could open the door to finding therapies to slow or potentially reverse the damage done by inflammation in the joint before the point of irreparable damage. Sedentary diabetic patients represent a target population that could benefit from early intervention, and future studies should assess how access to weight-bearing exercises may be used to recover joint function and prevent osteoarthritis progression.
